# Chronic kidney disease - determinants of progression and cardiovascular risk. PROGREDIR cohort study: design and methods: Doença renal crônica - determinantes de progressão e risco cardiovascular. Coorte PROGREDIR: desenho de estudo e métodos

**DOI:** 10.1590/1516-3180.2016.0272261116

**Published:** 2017-04-20

**Authors:** Maria Alice Muniz Domingos, Alessandra Carvalho Goulart, Paulo Andrade Lotufo, Isabela Judith Martins Benseñor, Silvia Maria de Oliveira Titan

**Affiliations:** I MD, PhD. Nephrologist, Renal Division, Department of Clinical Medicine, Faculdade de Medicina da Universidade de São Paulo (FMUSP), São Paulo (SP), Brazil.; II MD, PhD. Clinical Epidemiologist and Researcher, Clinical Research Center, University Hospital, Faculdade de Medicina da Universidade de São Paulo (FMUSP), São Paulo (SP), Brazil.; III MD, PhD. Full Professor, Clinical Research Center, University Hospital, Faculdade de Medicina da Universidade de São Paulo (FMUSP), São Paulo (SP), Brazil.; IV MD, PhD. Assistant Professor, Clinical Research Center, University Hospital, Faculdade de Medicina da Universidade de São Paulo (FMUSP), São Paulo (SP), Brazil.; V MD, PhD. Research Investigator, Renal Division, Department of Clinical Medicine, Faculdade de Medicina da Universidade de São Paulo (FMUSP), São Paulo (SP), Brazil.

**Keywords:** Renal insufficiency, chronic, Cardiovascular diseases, Renal replacement therapy, Biomarkers, Risk factors

## Abstract

**CONTEXT AND OBJECTIVE:**

Chronic kidney disease (CKD) has become an important public health issue. The socioeconomic burden of renal replacement therapy (RRT) is very high, as is CKD-related cardiovascular mortality and morbidity. Preventive and therapeutic measures only have modest impact and more research is needed. Few cohort studies have been conducted on populations with CKD. Our aim was to establish a cohort that would include more advanced forms of CKD (stages 3 and 4). Data collection was focused on renal and cardiovascular parameters.

**DESIGN AND SETTING:**

Prospective cohort study; São Paulo, Brazil.

**METHODS:**

Recruitment took place in Hospital das Clínicas, São Paulo, from March 2012 to December 2013. Data relating to medical history, food-frequency questionnaire, anthropometry, laboratory work-up, calcium score, echocardiography, carotid intimal-medial thickness, pulse-wave velocity, retinography and heart rate variability were collected. A biobank including serum, plasma, post-oral glucose tolerance test serum and plasma, urine (morning and 24-hour urine) and DNA was established.

**RESULTS:**

454 participants (60% men and 50% diabetics) of mean age 68 years were enrolled. Their mean estimated glomerular filtration rate-CKD Epidemiology Collaboration was 38 ml/min/1.73 m^2^. Follow-up is ongoing and the main outcomes are the start of RRT, cardiovascular events and death.

**CONCLUSIONS:**

The PROGREDIR cohort is a promising prospective study that will allow better understanding of CKD determinants and validation of candidate biomarkers for the risks of CKD progression and mortality.

## RESUMO

**CONTEXTO E OBJETIVO:**

A doença renal crônica (DRC) tornou-se um problema de saúde pública. A carga socioeconômica da terapia renal substitutiva é muito elevada, assim como a morbimortalidade cardiovascular associada à DRC. Medidas terapêuticas e preventivas têm impacto parcial e novos estudos são necessários. Há poucos estudos de coorte em populações com DRC. Nosso objetivo foi criar uma coorte que contemplasse formas mais avançadas de DRC (estágios 3 e 4). A coleta de dados foi centrada em parâmetros renais e cardiovasculares.

**TIPO DE ESTUDO E LOCAL:**

Estudo de coorte prospectivo; São Paulo, Brasil.

**MÉTODOS:**

O recrutamento ocorreu entre março de 2012 e dezembro de 2013, no Hospital das Clínicas, em São Paulo. Foram coletados dados de história médica, questionário de frequência alimentar, antropometria, exames laboratoriais, escore de cálcio, ecocardiografia, espessura de camada médio-intimal de carótidas, velocidade de onda de pulso, retinografia e variabilidade de frequência cardíaca. Um biobanco incluindo soro, plasma, soro e plasma pós-teste oral de tolerância à glicose, urina (manhã e 24 horas) e DNA foi estabelecido.

**RESULTADOS:**

454 participantes (60% homens e 50% diabéticos) com idade média de 68 anos foram recrutados. A taxa média de filtração glomerular estimada-Colaboração da Epidemiologia para DRC foi de 38,4 ml/min/1,73 m^2^. O seguimento está em andamento e os desfechos principais são: início de terapia renal substitutiva, eventos cardiovasculares e óbito.

**CONCLUSÃO:**

A coorte PROGREDIR é um estudo prospectivo promissor que permitirá melhor compreensão dos determinantes de DRC e a validação de biomarcadores candidatos para o risco de progressão de DRC e de mortalidade.

## INTRODUCTION

Chronic kidney disease (CKD) has become an important public health issue worldwide. Increasing prevalence of obesity and diabetes mellitus and today’s high life expectancy, particularly among patients with atherosclerosis, are all contributory factors. In addition, CKD progression is still a major challenge, with few new specific therapeutic measures available. The socioeconomic burden on individuals who need renal replacement therapy (RRT) is very high and comes together with CKD-related high cardiovascular mortality and morbidity, with incidence that may in fact even exceed the figures for RRT.^1^^,^^2^^,^^3^^,^^4^^,^^5^^,^^6^^,^^7^

In the United States, according to the Annual Report of the United States Renal Data System (USRDS),^8^ the prevalence of CKD stages 1-4 was around 14% in the general population and the incidence of end-stage renal disease (ESRD) was 353 cases per million/year in 2012. The prevalence of cardiovascular disease reached 69.8% among CKD patients versus 34.8% among individuals without CKD and the adjusted mortality rates for CKD patients was 76 deaths per 1000 patients, compared with 52 deaths per 1,000 individuals without CKD in 2012. Medicare expenses relating to CKD reach US$ 1700, 3500 and 12,700 per person-year for CKD patients with stages 2, 3 and 4, respectively.^9^ Overall, CKD accounts for 6.7% of total Medicare costs.^9^

In Brazil, there were 100,397 patients on dialysis at the end of 2013, with incidence of 170 cases per million/year and an estimated mortality rate of 17.9% per year.^10^ In 2013, 5,433 kidney transplantations were performed in Brazil, mostly using public resources.

Preventive measures are highly necessary, and the search for new biomarkers and new therapeutic strategies is intense. While several studies on general populations and cardiovascular cohorts have yielded important contributions towards CKD knowledge, more specific cohorts focusing on CKD progression instead of CKD incidence are necessary within nephrology. In response to this need, several countries like the United States (CRIC study), Germany (GCKD), Canada (CanPREDDICT), Japan, Australia and Uruguay, among others, have ongoing CKD cohort studies.^11^

Along the same lines, the PROGREDIR cohort study was designed to enable better understanding of the determinants of CKD progression and CKD-related mortality, with particular emphasis on mineral metabolism as a cardiovascular risk factor. The cohort comprises people with CKD stages 3 and 4 in São Paulo, Brazil. The cohort was established and baseline data were collected in 2012-2013. Prospective data on hard outcomes such as the start of renal replacement therapy, cardiovascular events and death are currently being gathered. The PROGREDIR cohort is funded by the Research Support Foundation of the State of São Paulo (Fundação de Amparo à Pesquisa do Estado de São Paulo, FAPESP; 2011-17341-0), São Paulo, Brazil.

## OBJECTIVE

The aim of this study was to establish a CKD cohort that would include participants with more advanced forms of this disease (CKD stages 3 and 4), with data collection focused on renal and cardiovascular parameters.

## METHODS

### Study population and recruitment

Patients attending the outpatient service of Hospital das Clínicas, São Paulo, a public university facility providing quaternary-level care for patients with chronic diseases, were invited to participate in this study. Initially, from the outpatient records, all patients aged *≥* 30 years and at least two measurements of creatinine (with a minimum interval of 3 months) *≥* 1.6 mg/dl for men and *≥* 1.4 mg/dl for women were considered potential candidates. Patients attending oncology, psychiatry, human immunodeficiency virus/acquired immunodeficiency syndrome (HIV/AIDS), viral hepatitis and glomerulonephritis services were excluded. The remaining candidates were then contacted by phone and were invited to participate if they did not meet any exclusion criteria. The exclusion criteria checked by the interviewer were: hospitalization within the last six months, acute myocardial infarction within the last six months, autoimmune diseases, pregnancy, psychiatric diseases, ongoing chemotherapy or immunosuppressive therapy, ongoing RRT, glomerulonephritis, HIV/AIDS infection, hepatitis B or C and any organ transplantation. Recruitment took place between March 2012 and December 2013, and 454 participants were enrolled. The study was approved by two local ethics committees and written informed consent was obtained from all participants.

### Sample size estimation

The sample size was calculated using an estimate of the annual incidence of end-stage renal disease (ESRD) of 2% and an annual rate of cardiovascular events of 2-3.5% among diabetic nephropathy patients.^12^ By assuming a difference in event rate incidence of 3% between exposed and non-exposed subjects, a sample size of 500 was estimated, with an alpha error of 0.05 and a power of 80%.

### Baseline examination and data collection

The baseline assessment lasted approximately six hours and was performed on a single-day visit to our study center. Data collection included all the variables depicted in Table 1. Sex and self-declared race were also registered. Anthropometry was performed first, with the participants wearing light clothes, following standard techniques.^13^ Blood pressure (BP) was measured using a validated oscillometric device (Omron HEM 705CPINT). Three measurements were made at one-minute intervals. The mean of the last two BP measurements was used as the definition for high blood pressure. Overnight fasting blood samples and 24-hour and spot urine samples were collected. A standard 75-g oral glucose tolerance test was administered to all participants without known diabetes. Urine and blood aliquots were prepared and stored at -180 °C in nitrogen. DNA extraction was performed and the material was stored at -80 °C. Baseline laboratory measurements were made using conventional techniques (Table 2).

**Table 1:**
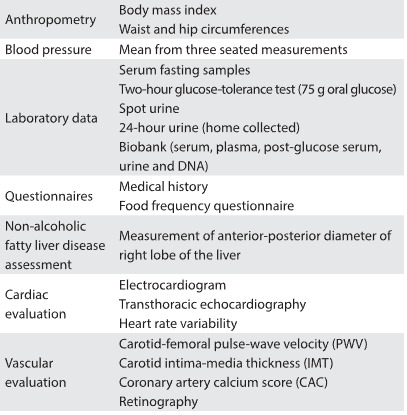
Baseline assessments in PROGREDIR cohort study

**Table 2:**
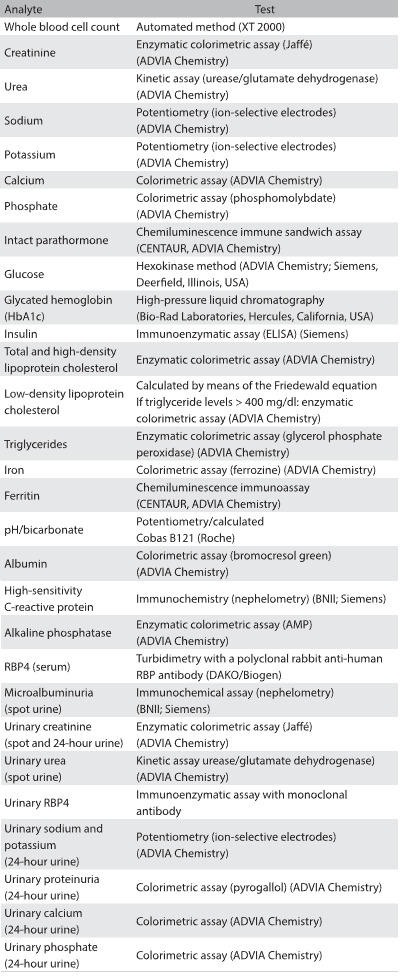
Baseline laboratory tests

Interviews were conducted by trained personal under strict quality control. Data on medical history, socioeconomic variables, family history, medication use, physical activity, smoking and alcohol consumption were obtained. A food-frequency questionnaire was also administered. The food list was defined on the basis of the dietary intake of the Brazilian population and the reproducibility and validity of the questionnaire has been measured elsewhere.^14^

Conventional 12-lead electrocardiograms (ECG) were performed using a digital device (Atria 6100, Burdick, Cardiac Science Corporation, USA) with automated readings of heart rate; P wave, QRS complex and T wave duration, amplitude and axis; and QT, QTc and QT dispersion. All precordial electrodes were positioned after identifying the location for the V4 electrode with a square. The Electrocardiogram Reading Center (ERC) at the Heart Institute of the University of São Paulo (INCOR) provided all ECG readings.

For heart rate variability determinations, a 10-minute continuous ECG was obtained from a single lead (usually D2) using a digital electrocardiograph (Micromed, Brazil) at a frequency of 250 Hz, with subjects in the supine position. Computer software (WinCardio) was used to generate time series of RR intervals that were sent to the central cardiovascular physiology laboratory (IC-ES). All readings were made through computer software that eliminated artifacts and selected RR intervals lasting 0.5 to 2.0 seconds. Temporal and spectral analyses of heart rate variability (HRV) were then performed using an autoregressive model to identify very low-frequency (VLF, 0 to 0.04 Hz), low-frequency (LF, 0.04 to 0.1 Hz) and high-frequency spectral bands (HF, 0.1 to 0.4 Hz).

Transthoracic echocardiography was performed on all participants using a device (Aplio XG; Toshiba Corporation, Tokyo, Japan) with a 2.5 MHz sector transducer. All examinations were performed by the same echocardiographer. The readings consisted of qualitative analysis of echocardiographic findings and measurements of quantitative parameters such as: left ventricular (LV) geometry and size, left atrial size, LV systolic and diastolic function, segmental LV dysfunction, valvular heart disease and pericardial appearance. Cardiac mass was calculated using the Devereaux formula.^15^

Measurement of the anterior-posterior diameter of the right lobe of the liver was performed by means of ultrasound for quantitative assessment of nonalcoholic fatty liver disease (NAFLD). Liver images were obtained using standard equipment (Toshiba SSA-770A Aplio, Japan) and a broadband convex transducer (PVT-375BT) with a central frequency of 3.5 MHz (2.5-5.5 MHz).^16^

The carotid-femoral pulse-wave velocity (PWV) was measured using a validated automated device (Complior, Artech Medicale, France), with the subject in the supine position in a temperature-controlled room (20-24 °C). First, BP was measured in the right arm with the subject in the supine position using an oscillometric device (HRM Onrom 705 CP). The distance from the sternal furcula to the right femoral pulse was determined using a measuring tape regardless of abdominal curvature. Pulse sensors were positioned in the right carotid and femoral arteries so that pulse waves were recorded and viewed on a computer screen. Computer software that could adequately detect and record pulse waves was used. PWV was calculated by dividing the distance from the sterna furcula to the femoral pulse by the difference between the rise delays of the carotid and femoral pulses. A subject’s PWV was the arithmetic average of readings obtained in ten consecutive cardiac cycles at a regular heart rate.

Carotid intimal-media thickness (IMT) was assessed in all patients in a standardized manner using a device (Aplio XG, Toshiba) with a 7.5 MHz linear transducer. The technique used for IMT measurement was as previously published.^17^ IMT was measured in the outer wall of a predefined carotid segment of 1 cm in length from 1 cm below the carotid bifurcation, during three cardiac cycles. We considered the images acquired to be valid if they clearly showed three reference points on both sides:

1. anatomical guides for the common carotid arteries;
2. interfaces between the lumen and the far wall of the vessel; and
3. interfaces between the media and adventitia layers of the far wall of the vessel.

We used the MIA software to standardize the readings and interpret the carotid scans as previously described. IMT was then defined as the mean of the right and left carotid measurements.

To determine the coronary artery calcium score, the participants underwent non-contrast computed tomography. The scans were performed using a 64-slice detector computed tomography scanner (Philips Brilliance, Philips, Netherlands). After scout images had been produced, each patient also underwent an ECG-gated prospective calcium score examination with a tube potential of 120 kV and a tube current adjusted to body habitus. Images were reconstructed at 2.5 mm slice thickness using standard filtered back projection. The coronary artery calcium score was expressed in terms of Agatston units and the percentiles were evaluated in a blinded manner by an experienced cardiologist using semi-automated software (Calcium Scoring, Philips Workstation). Coronary calcium scores were not obtained for participants who reported that they had been fitted with coronary stents, since the stent material greatly overestimates the calcium scores.

Retinography was performed using a nonmydriatic retinograph (CR-1, Canon, Japan) with a 10-megapixel digital camera (Canon EOS 40 D). The subjects underwent natural dilation of their pupils through resting in a darkened room for about four minutes, and for each eye two 45° fundus images were obtained: one centered on the optical disk and the second on the maculae. Our institution’s central retinography laboratory (IC-RS) developed standardized image acquisition and reading protocols, and DICOM images (approximately 30 MB) and JPEG images (approximately 3 MB) were acquired. The JPEG images were recorded on CD/DVD at the study sites and were mailed to the central retinography laboratory.

### Follow-up

The participants are being contacted again annually, for telephone interviews that include questions on hospitalizations, need for RRT and self-rated health. The main clinical endpoints investigated are death, acute myocardial infarction, unstable angina pectoris, cardiac revascularization, heart failure, stroke and RRT. Any cardiovascular and renal clinical events that are reported are then investigated and classified in line with the study protocol, by a panel of physicians that has received training in accordance with international classification criteria.^18^ In the event of the participant’s death, information regarding this event is sought. Surveillance of clinical events is also conducted through state databases such as the Mortality Registry and the São Paulo State Registry of Dialysis and Transplantation.

## RESULTS

Over the two-year recruitment period, 454 participants were enrolled. Table 3 shows the main clinical and laboratory parameters at baseline. The population recruited mainly had CKD in stages 3 and 4, with a mean estimated glomerular filtration rate-CKD Epidemiology Collaboration (eGFR-CKDEPI) of 38.4 (± 14.6) ml/min/1.73 m^2^. The albuminuria range was wide, with similar frequencies of normoalbuminuria (35%), microalbuminuria (31%) and macroalbuminuria (34%). The participants’ median age was 67 years; 63% were men; 60% were current or past smokers; 45% self-reported diabetes; and 32% reported having had previous myocardial infarction. Coronary artery calcification scores were also high, with more than half of the cohort presenting an Agatston score above 100.

Follow-up is ongoing. Up to the present date, i.e. over the first three years of follow-up, event rates have been high, with a 5-7% mortality rate per year and 2-3% incidence of ESRD and non-fatal cardiovascular events per year. With this event rate, from 2017 onwards, survival analysis will be started, focusing on biomarkers for mineral metabolism.

**Table 3:**
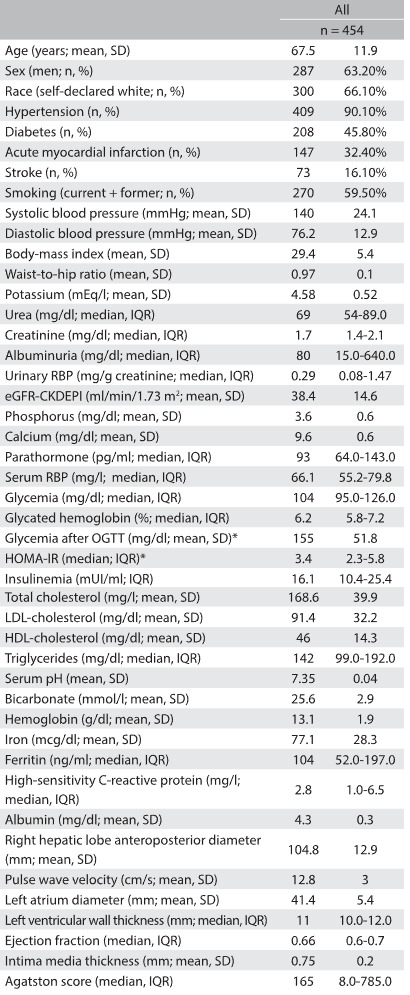
Baseline clinical and laboratory profile of 454 participants in the PROGREDIR cohort

*calculated for participants without known diabetes. SD = standard deviation; IQR = interquartile range; RBP = retinol-binding protein; eGFR-CKDEPI = estimated glomerular filtration rate-Chronic Kidney Disease Epidemiology Collaboration; OGTT = oral glucose tolerance test; HOMA-IR = homeostasis model assessment as an index of insulin resistance; LDL = low-density lipoprotein; HDL = high-density lipoprotein.

## DISCUSSION

The PROGREDIR cohort was designed specifically to address CKD progression among patients with moderate to advanced disease. Over a two-year recruitment period, we were able to enroll 454 participants, and thus nearly reached the estimated sample size. The baseline characteristics of these participants were in accordance with the profile expected from the inclusion and exclusion criteria: older age, predominance of men and high rates of diabetes and previous cardiovascular disease. In the PROGREDIR cohort, we avoided overrepresentation of glomerulonephritis and other specific kidney diseases such as those relating to HIV, hepatitis C and lupus. Transplantation patients (any organ) were also not included. This decision was mostly related to the fact that PROGREDIR was designed to be a cohort of general CKD cases and not to address mechanisms relating to specific systemic or primary diseases. The eligible participants were originally from a quaternary-level hospital, which might have yielded an excessive number of glomerulonephritis cases if exclusion criteria had not been applied. Other CKD cohort studies have applied similar inclusion and exclusion criteria and have ended up with recruited populations compatible with the profile observed in PROGREDIR.^19^^,^^20^^,^^21^

One important accomplishment was to have nearly equal representation of normoalbuminuria, microalbuminuria and macroalbuminuria subpopulations in the baseline profile of the cohort. The prevalence of and interest in normoalbuminuric CKD is increasing,^22^^,^^23^^,^^24^ since it is now known that 30-45% of diabetic patients may in fact present CKD and normoalbuminuria. It is currently of interest not only to understand the determinants of CKD progression in the normoalbuminuric CKD population, but also to compare the performance of traditional and new risk factors in normoalbuminuric and albuminuric populations, in order to test whether the results can be generalized to a broad spectrum of diseases.

Baseline data were collected in this study in accordance with the study design, covering traditional cardiovascular risk factors and biomarkers for CKD. Surrogate measurements of atherosclerosis and hypertension such as coronary calcium score, cardiac hypertrophy, IMT, PWV and retinography were made, and these will allow understanding and stratification of baseline CKD among the participants. The biobank is wide-ranging and kept under strict quality control, thus providing a source for reliable future measurements.

Follow-up is ongoing and a high event rate is being observed. Follow-up data collection is being centered on three major clinical events: death, non-fatal cardiovascular events and starting of RRT. These events are of particular importance, since CKD is known to be a very important cardiovascular risk factor that makes a significant contribution to high rates of morbidity and mortality.^2^ Focusing data collection only on renal events would lead to selection bias, because a significant proportion of the participants might experience cardiovascular events prior to renal events. Thus, to fully address the impact of CKD biomarkers and measurements, it is very important to account for their impact both on renal events such as mortality and on fatal and non-fatal cardiovascular events.

Now that the cohort has been established, the PROGREDIR study can be used for research investigation in two major ways. First, it can be used to test the performance of candidate biomarkers for CKD progression. The current need to promote discovery and validation of biomarkers in CKD is highlighted by the recent launch of a CKD Biomarkers Consortium (BioCon)^25^ by the National Institute of Diabetes and Digestive and Kidney Diseases in the United States. Similar approaches are being used by European countries.^26^ Secondly, the cohort can be used to test high throughput technologies, which are an innovative approach that may provide new insights on the mechanisms and pathways of complex diseases, as well as enabling identification of novel biomarkers for diseases. As a first step, untargeted metabolomic assessments are currently being performed on baseline serum and the data thus obtained will be analyzed in relation to renal function and clinical events.

Additionally, to contribute towards improvement of scientific knowledge on CKD, the PROGREDIR study will also serve the purpose of being a national data source in which biomarkers can be replicated and validated. Racial factors are known to have an important effect on the risk of diseases, and this has recently been very well illustrated by the discovery of the higher risk attributable to the APOL1 gene in the African-American population.^27^ In this regard, it is very important that national datasets should be available, so that the performance of candidate biomarkers can be tested on the Brazilian population, which is known to be highly admixed.

## CONCLUSION

In conclusion, the PROGREDIR cohort recruitment and baseline data collection were successfully implemented. In addition to being a national dataset, the PROGREDIR cohort provides promising prospective study material that will allow better understanding of CKD determinants and validation of candidate biomarkers for CKD progression and mortality risk.
